# A case of early gastric cancer with a single giant lymph node metastasis

**DOI:** 10.1186/s40792-021-01328-y

**Published:** 2021-11-18

**Authors:** Masato Yoshikawa, Misaki Tamario, Masayoshi Obatake, Koichi Sato, Shigehiko Yagi, Hiromi Otani, Katsumi Kito

**Affiliations:** 1 Institute of Ehime Prefectural Central Hospital, Department of Digestive Surgery, Matsuyama, Ehime 790-0024 Japan; 2 Department of Pathology, Ehime Prefectural Central Hospital, Matsuyama, 790-0024 Japan

**Keywords:** Early gastric cancer, Giant lymph node metastasis, Retrodifferentiation, Yolk sac tumor-like carcinoma

## Abstract

**Background:**

Early gastric cancer (EGC) is often associated with lymphatic metastasis, but it is extremely rare to be found as a single giant lymph node. Cancer often becomes more malignant in metastatic lesions than in primary lesions, and retrodifferentiation to the fetal gastrointestinal tract during the metastatic process has been reported in gastric cancer. We report an extremely rare case of EGC with a 13-cm giant lymph node metastasis in which an adenocarcinoma with enteroblastic differentiation and yolk sac tumor-like components was observed.

**Case presentation:**

The case was a 70-year-old man who visited his local doctor with right hypochondrial pain, which was identified by computed tomography (CT) as a giant mass. Upper endoscopy revealed a 30-mm-sized 0-IIc lesion in the greater curvature of the angular incisure and a 15-mm-sized 0-IIa lesion in the anterior wall of the lower body of the gastric body. Endoscopic biopsy revealed tubular adenocarcinoma in both lesions. The gastric lesion and the giant tumor were clinically regarded as independent lesions (gastrointestinal stromal tumor, [GIST], and EGCs), and distal gastrectomy and D1 + dissection were performed to comprehensively treat all lesions. Pathological examination revealed that the giant tumor was tubular adenocarcinoma with an intestinal phenotype and was considered a lymph node metastasis of EGCs. To exclude the possibility of metastasis of adenocarcinoma other than EGCs, postoperative positron emission tomography-computed tomography (PET-CT) and colonoscopy were performed; however, no primary site other than the stomach was found. Metastatic lymph nodes have an increased degree of atypia compared with the primary tumor, and yolk sac tumor-like carcinoma morphology was observed along with α-fetoprotein (AFP) and Spalt-like 4 (SALL4) expression in this case. It was considered that retrodifferentiation to a fetal phenotype occurred during the metastatic process. Liver metastasis occurred 6 months after surgery, and chemotherapy is currently being introduced.

**Conclusions:**

We experienced a case of EGC with a single giant lymph node metastasis. Retrodifferentiation to the fetal gastrointestinal tract during metastasis was speculated to be involved in the formation of giant lymph node metastasis and liver metastasis in this case.

## Background

The frequency of lymph node metastasis in early gastric cancer (EGC) is approximately 16.9% in submucosal layer (SM) cancer [1]. However, EGC with a single giant lymph node metastasis of more than 5 cm is extremely rare. It is difficult to diagnose lymph node metastasis preoperatively, and in past reports, there have been no cases that giant tumor was diagnosed as lymph node metastasis from EGC, and many cases were diagnosed as gastric submucosal tumors or pancreatic tumors [2]. The phenomenon that cancer becomes more malignant in metastatic lesions than in primary lesions is often experienced, and retrodifferentiation to the fetal gastrointestinal tract during the metastatic process also occurs in gastric cancer [3, 4]. In this case, we experienced a case with a 13 cm single giant lymph node metastasis of EGC. We report here an extremely rare case of EGCs with a giant lymph node metastasis in which adenocarcinoma with enteroblastic differentiation and yolk sac tumor-like component was observed.

## Case presentation

**Fig. 1:**
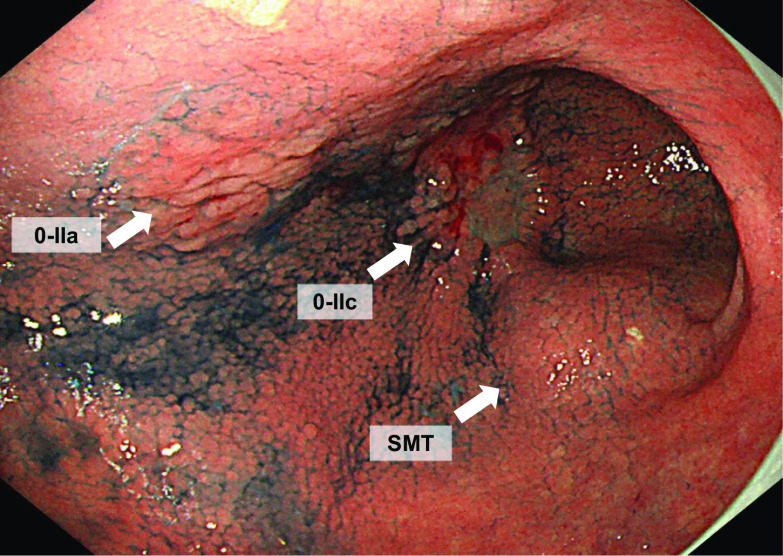
Upper endoscopy: the 0-IIc lesion, the 0-IIa lesion and the SMT like lesions (arrows)

A 70-year-old man visited his doctor because of right hypochondrial pain, and a giant tumor in the abdomen was identified on computed tomography (CT). In addition, two EGCs were revealed by upper endoscopy, and he was referred to our department. His medical history was type 2 diabetes mellitus and postoperative state of appendectomy, and he had no family history. His height was 158 cm, his weight was 59.7 kg, and his BMI was 23.9, and he had a fist-sized palpable elastic hard mass on the right abdomen. Laboratory tests showed low levels of albumin (3.4 g/dL) and hemoglobin (10.6 g/dL), and high levels of carcinoembryonic antigen (CEA) (18.9 ng/mL). Upper endoscopy revealed a 30-mm-sized 0-IIc lesion in the anterior wall of the angular incisure and a 15-mm-sized 0-IIa lesion in the lower anterior wall of the gastric corpus (both tub2). A submucosal tumor-like ridge in the lower body of the stomach was suspected to be an exclusion by the aforementioned giant tumor (Fig. 1).

**Fig. 2:**
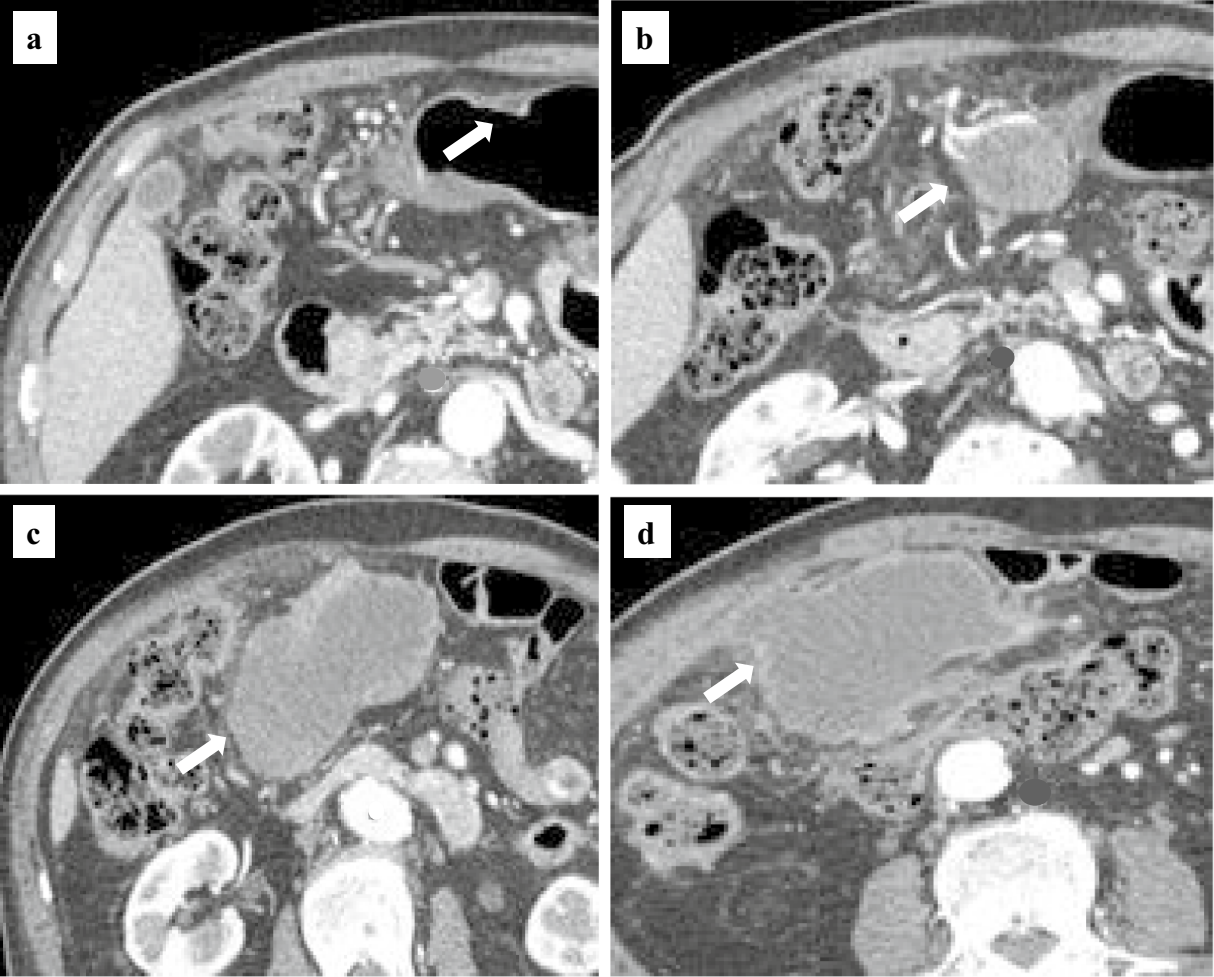
Abdominal enhanced CT: the 0-IIc lesion (**a**) and the giant tumor (**b**, **c**, **d**)

**Fig. 3:**
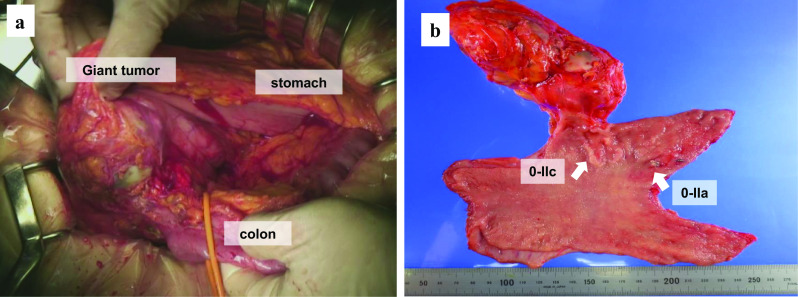
Intraoperative findings (**a**) and macroscopic findings (**b**)

Abdominal enhanced CT confirmed the curvatures of the anterior wall of the angular incisure as a thickening of the wall with a contrast effect. Lesions in the lower body of the stomach could not be identified. The abdominal mass was recognized as a malformed tumor with a maximum diameter of 13 cm, and with a solid component that was lightly contrast-enhanced in its interior, which was located outside the stomach wall on the greater curvature side of the angular incisure. The boundary with the abdominal wall was unclear, and infiltration of the abdominal wall was suspected (Fig. 2). The preoperative diagnosis was GIST and two EGCs (M, Ant, 0-IIc, cT1bN0M0 cStage I; M, Ant, 0-IIa, cT1aN0M0 cStage I). Distal gastrectomy, D1 + lymph node dissection, and cholecystectomy were performed for the diagnosis of gastric GIST and EGCs. Reconstruction was performed by Billroth I. The giant tumor was identified as a large fist-shaped mass located in the greater curvature of the angular incisure that was adhered to the transverse colon and the transverse mesentery (Fig. 3a). The surgery time was 3 h and 45 min, and the blood loss volume was 232 ml. Although the preoperative and intraoperative diagnosis was GIST, macroscopic findings of the resected specimen showed there was no continuity between the giant tumor and the stomach wall (Fig. 3b).

**Fig. 4:**
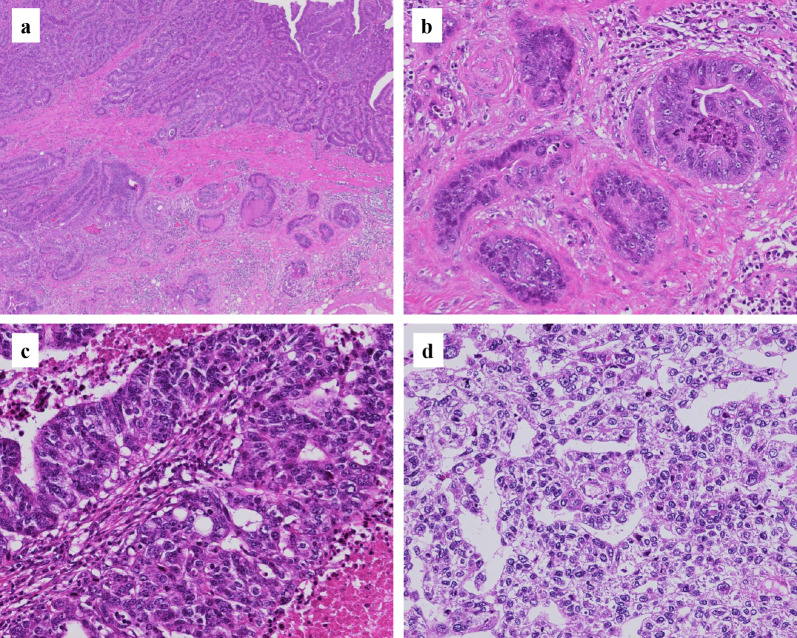
Hematoxylin and eosin stain: the gastric type 0-IIc lesion showed a tubular adenocarcinoma infiltrating into the submucosal layer (**a**, **b**). The metastatic adenocarcinoma with necrosis in the giant lymph node (**c**). The metastatic adenocarcinoma contained yolk sac tumor-like components with Schiller-Duval bodies (**d**) (original magnification, **a** ×40, **b**–**d** ×200)

**Fig. 5:**
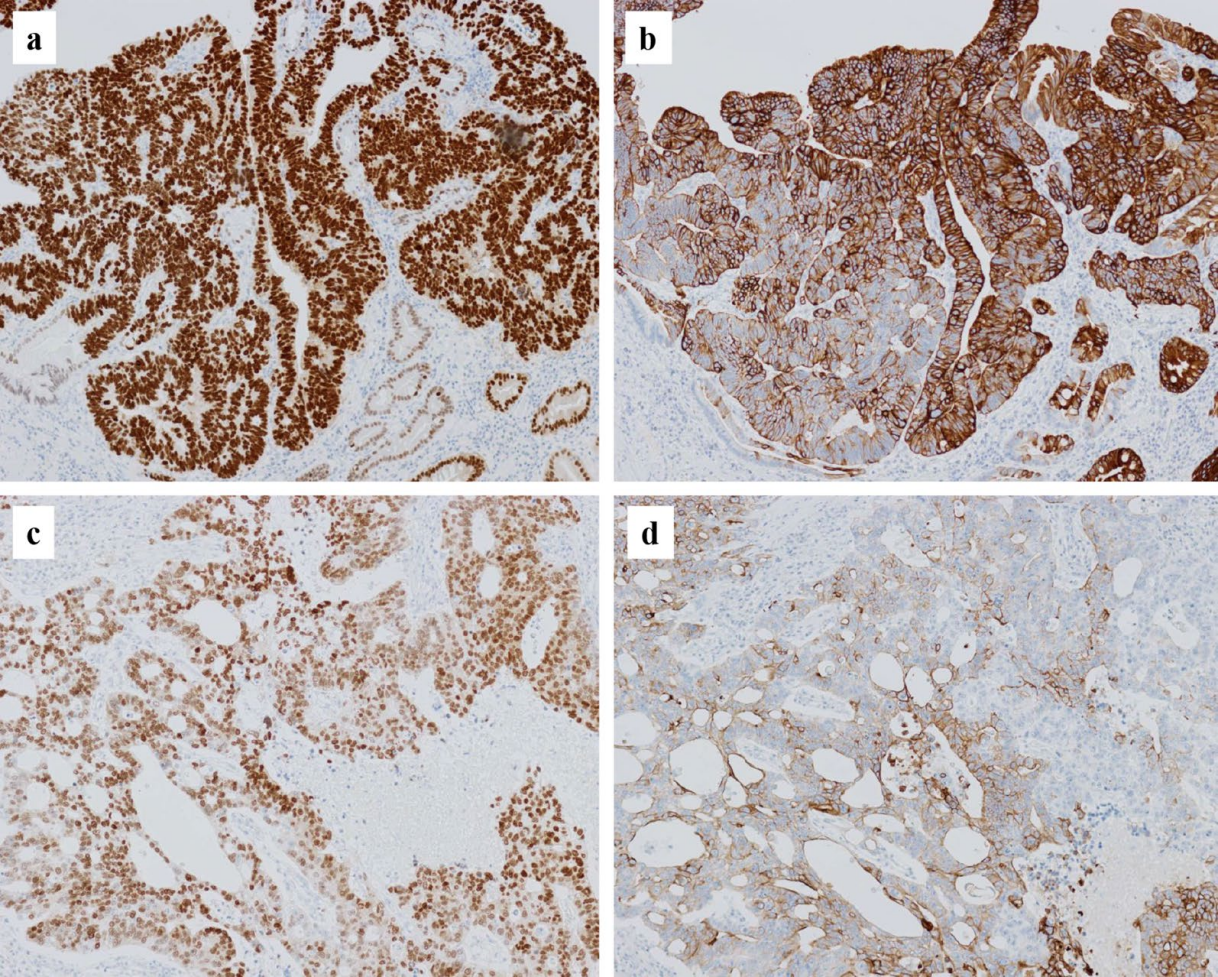
Immunohistochemical staining of CDX-2 and CK20: CDX-2 expression of the 0-IIc lesion (**a**), and the giant tumor (**c**). CK20 expression of the 0-IIc lesion (**b**), and the giant tumor (**d**) (original magnification, each × 100)

**Fig. 6:**
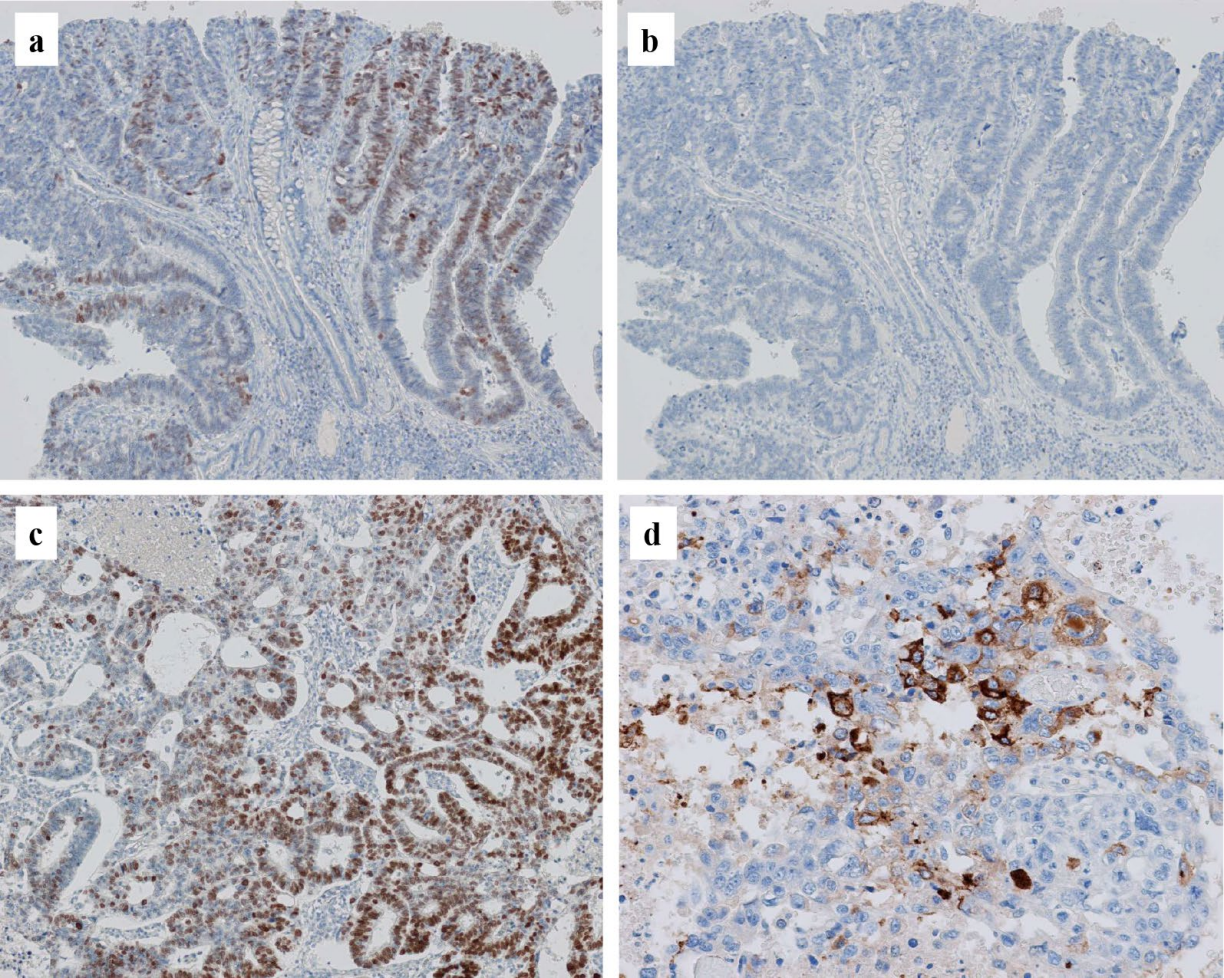
Immunohistochemical staining of SALL4 and AFP: SALL4 expression of the 0-IIc lesion (**a**), and the giant tumor (**c**). AFP expression of the 0-IIc lesion (**b**), and the giant tumor (**d**) (original magnification, **a**: ×40, **b**: ×40, **c**: ×100, **d**: ×200)

Histopathological findings are shown in Figs. 4, 5 and 6. The gastric lesions were diagnosed as two primary EGCs (M, Ant, type 0-IIc, 32 × 30 mm, tub1 > tub2, pT1b (SM2), INFb, Ly1a, V1a, CY0, N1 (1/40), M0, pStage IB; and M, Ant, type 0-IIa + IIc, 10 × 10 mm, tub1, pT1a (M), Ly0, V0, CY0, N0, M0, pStage IA), and their histopathology was conventional tubular adenocarcinoma (Fig. 4a, b). The smaller lesion (type 0-IIa) was an intramucosal well-differentiated adenocarcinoma, while the larger one (type 0-IIc) infiltrated into a submucosal layer with lymphovascular invasion. The extragastric giant tumor was a moderately differentiated tubular adenocarcinoma with hemorrhage and extensive necrosis (Fig. 4c). Immunohistochemically, caudal type homeobox-2 (CDX-2) and cytokeratin 20 (CK20), representing an intestinal phenotype, were positive in both the gastric lesions and the giant tumor (Fig. 5). In addition, enteroblastic differentiation and a yolk sac tumor-like components with Schiller–Duval bodies were observed in the giant tumor. Approximately half of the cancer cells in the giant tumor were immunoreactive for SALL4 and some were AFP-positive (Fig. 6). Both human chorionic gonadotropin (β-hCG) and synaptophysin were negative. Except for the giant lymph node metastasis, no metastasis was observed in the other 39 dissected lymph nodes. Because the lymph node metastasis was solitary and large, a whole-body search was performed by colonoscopy and PET-CT during the early postoperative period to detect other primary sites; however, there was no primary site other than the stomach. Therefore, we concluded that the giant tumor containing carcinoma with enteroblastic differentiation was a lymph node metastasis from the EGC.

**Fig. 7:**
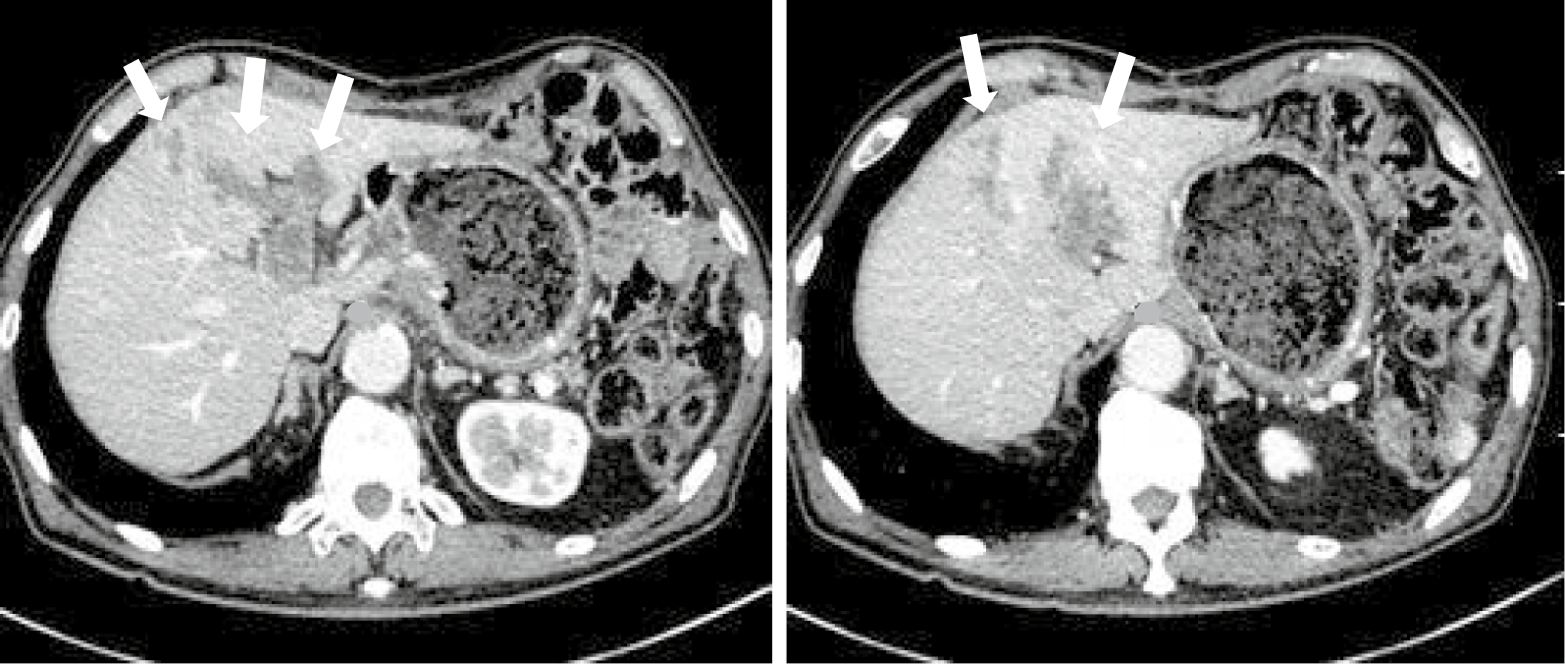
Abdominal enhanced CT of 6 months after surgery: metastatic lesions are indicated by arrows

Postoperative delayed gastric excretion occurred (Clavien–Dindo classification II), and decompression by gastric tube was need for 5 days. The patient was discharged on postoperative day 16. Postoperative adjuvant chemotherapy was not performed, and the patient was followed up. However, 6 months after surgery, his markers had risen. CT showed liver metastasis with a portal vein tumor plug, and SOX (S-1 + oxaliplatin) therapy was started (Fig. 7). The patient is alive 1 year after the surgery with stable disease (RECIST guideline version 1.1).

## Discussion

We experienced an extremely rare case of single giant lymph node metastasis from EGC. Pathological findings showed that the giant lymph node metastasis was moderately differentiated adenocarcinoma similar to that of primary EGC, and an intestinal phenotype was observed in both the primary and metastatic lesions by immunostaining. Because no primary lesion other than the stomach could be identified by postoperative colonoscopy and PET-CT examination, the giant tumor was diagnosed as lymph node metastasis of EGC.

We diagnosed the giant tumor as GIST only by CT and upper endoscopy. PET-CT was not considered due to poor glycemic control. MRI was not also considered for the definitive diagnosis of gastric submucosal tumor because of its lack of specificity in signal intensity and its pattern [5]. Because of concern regarding dissemination by needle biopsy, surgery was performed without biopsy.

In this case, we diagnosed EGC and GIST, and performed D1 + lymphnode dissection.

The decision was based on the idea that lymph node dissection was not necessary for GIST unless lymph node metastasis were apparent [6]. We determined that the giant tumor connected to the stomach wall and identified it as GIST from the preoperative and intraoperative findings. However macroscopic findings of the resected specimen showed there was no connection between the giant tumor and the stomach wall. It was suspected that the giant tumor could be an isolated tumor such as lymph node metastasis or primary omentum tumor. As a result, we could have decided the appropriate lymph node dissection range (D2 lymph node dissection) by frozen section during operation.

Four cases of EGC with a single giant lymph node metastasis of 5 cm or more were previously reported (Table 1), and in most cases the main complaint was abdominal symptoms resulting from the giant lymph node. No cases have been diagnosed as lymph node metastasis of gastric cancer before surgery, and diagnoses of pancreatic head, liver, and gastric submucosal tumors have been made. All cases were performed gastrectomy + lymph node dissection and alive without recurrence, although the follow-up period was shorter than 5 years [2, 7–9].

**Table 1:** Previous reports of EGC with a single giant lymph node metastasis

Year	Writer	Age/sex	Chiefcomplaint	Preoperativediagnosis	Postoperative diagnosis (differentiation, depth, size: cm)	Procedure	LN	Adjuvant chemo	Prognosis
1987	Kamiya et al.	66/M	Upper abdominal pain	Hepaticcyst adenocarcinoma	L, Less (tub1, m,0.8) L,Ant (tub1, m,0.4)	Partial gastrectomy	#5 5 cm	–	2Y1M Norec
1996	Terashima et al.	59/F	Abdominalmass	Gastriccancer and Pancreas or Liver tumor	M, Less (por, sm, −)	Partial gastrectomy, LateralHx, DP, Splenectomy	#3 14 cm	–	4Y0M No rec
2009	Ishii et al.	57/M	-	Gastriccancer and Pancreas tumor	L,Post (tub1,sm, 2.5) L, Post (tub1,sm, 0.7) L,Ant (tub1-por1, sm, 0.6)	DG, D1+	#6 6 cm	Unknown	1Y10M No rec
2019	Umeda et al.	72/M	Abdominalmass	SMT	M, Less (tub1-2,mp, 2.5)	DG, D2	#6 8 cm	+ Regimen:unknown	1Y8M No rec
2021	Our case	78/M	Abdominalmass	SMT	M, Ant (tub1,sm, 3.0) M, Ant (tub1,sm, 1.5)	DG, D1+	#4d 13 cm	–	6 M Liver meta → SOX

The formation of large lymph nodes causes obstruction of lymph vessels and stagnation of lymph flow, and when cancer cells grow abnormally in one lymph node and most of the lymph nodes are occupied by lesions, it is believed that lymph flow is blocked and metastasis to the periphery is impeded. Therefore, it is thought that a good prognosis can be obtained by excision [10].

However, in this case, although it was a single lymph node metastasis, hematogenous metastasis to the liver occurred as early as 6 months after surgery. In the pathological diagnosis, the degree of atypia increased in the metastatic lymph nodes compared with the primary lesion, and the metastatic lymph nodes had a clear cytoplasm, a Schiller–Duval body-like structure that was not found in the primary lesion, and a yolk sac tumor-like carcinoma morphology. To date, retrodifferentiation to the yolk sac tumor or embryonic gastrointestinal tract during metastasis has been presumed to be the mechanism by which gastric cancer acquires fetal features and has not been found in the primary lesions with metastatic characteristics [3, 4]. In our case, retrodifferentiation to a yolk sac tumor during metastasis was considered a factor in the increased tumor malignancy.

In addition, in this case, the expression of SALL4, a fetal cancer protein, was observed in the primary lesion, although AFP expression was not observed. Recently, in addition to AFP, glypican-3 and SALL4 have been considered effective in diagnosing AFP-producing gastric cancer as fetal cancer proteins. Ushiku et al. reported that AFP production was observed in 8% of 338 cases of gastric cancer, and that all AFP-positive cases were also positive for glypican-3 and SALL4. Furthermore, in a study of 32 cases of AFP-producing gastric cancer, only 16% were positive for diffuse staining (50% or more of tumor cells) in AFP, whereas it was as high as 56% positive for glypican-3 and 78% for SALL4 [11]. In this case, although AFP expression and typical histology of yolk sac tumors were not observed in the primary lesion, SALL4 expression was positive, and the tumor had the characteristics of a fetal tumor. The most characteristic feature of gastric cancer with fetal components is that it is highly vascularly invasive and prone to liver metastasis. In a comparative study of fetal gastrointestinal epithelial-like cancer and conventional gastric cancer, lymphatic invasion (76% vs 41%), venous invasion (72% vs 31%), lymph node metastasis (69% vs 38%), and liver metastasis (31% vs 6%) were all significantly more frequent in carcinoma with enteroblastic differentiation. Regarding the prognosis, the 1-year survival rate was 38.7%, and was reported as 66.7% even in curative resection cases; thus, the prognosis is extremely poor compared with common typed gastric cancer [12]. In the current case, liver metastasis was observed early within 1 year after surgery and postoperative chemotherapy was introduced. Strict postoperative follow-up is essential for gastric cancer with fetal components, and, even in cases that could be resected, postoperative adjuvant chemotherapy that is stronger than that for common typed gastric cancer may be considered.

## Conclusions

We experienced a case of EGC with a single giant lymph node metastasis. Retrodifferentiation to the fetal gastrointestinal tract during metastasis was considered to be involved in the formation of giant lymph node metastasis and liver metastasis in this case.

## Data Availability

The authors declare that all data in this article are available within the article.

## Abbreviations

EGC: Early gastric cancer
CT: Computed tomography
GIST: Gastrointestinal stromal tumor
PET-CT: Positron emission tomography-computed tomography
AFP: α-Fetoprotein
SALL4: Spalt-like 4
SM: Submucosal layer
CEA: Carcinoembryonic antigen
LN: Lymph node
CDX-2: Caudal type homeobox-2
CK20: Cytokeratin 20
β-hCG: Human chorionic gonadotropin
SOX: S-1 + oxaliplatin
Hx: Hepatectomy
DP: Distal pancreatectomy
DG: Distal gastrectomy
